# Polyphosphazenes: Multifunctional, Biodegradable Vehicles for Drug and Gene Delivery

**DOI:** 10.3390/polym5010161

**Published:** 2013-03-01

**Authors:** Ian Teasdale, Oliver Brüggemann

**Affiliations:** Institute of Polymer Chemistry, Johannes Kepler University, 4060, Leonding, Austria; oliver.brueggemann@jku.at

**Keywords:** polyphosphazenes, biodegradable polymers, drug delivery, gene delivery, polymer therapeutics, nanomedicines

## Abstract

Poly[(organo)phosphazenes] are a unique class of extremely versatile polymers with a range of applications including tissue engineering and drug delivery, as hydrogels, shape memory polymers and as stimuli responsive materials. This review aims to divulge the basic principles of designing polyphosphazenes for drug and gene delivery and portray the huge potential of these extremely versatile materials for such applications. Polyphosphazenes offer a number of distinct advantages as carriers for bioconjugates; alongside their completely degradable backbone, to non-toxic degradation products, they possess an inherently and uniquely high functionality and, thanks to recent advances in their polymer chemistry, can be prepared with controlled molecular weights and narrow polydispersities, as well as self-assembled supra-molecular structures. Importantly, the rate of degradation/hydrolysis of the polymers can be carefully tuned to suit the desired application. In this review we detail the recent developments in the chemistry of polyphosphazenes, relevant to drug and gene delivery and describe recent investigations into their application in this field.

## 1. Polymeric Carriers for Drug Delivery

In recent years, the use of polymers for nanomedicine has gained much attention in medicine and biology (reviewed in [1]). A vast array of materials have been designed, prepared and tested, ranging from conventional drug-delivery formulations that entrap and/or solubilize drugs and matrices for controlled drug release, through to supramolecular systems and multi-component polymer based drugs [2]. Examples include: polymer-drug conjugates [3], dendritic and hyperbranched carriers [4], polymer-antibody conjugates [5], polymer micelles and polymersomes [6-8], nanospheres and capsules [9]. Such systems are often referred to as “polymer therapeutics” [4], although this term is reserved by some authors for those systems in which the drug is chemically conjugated to the polymeric carrier [10]. A common feature of such materials is that they modify the pharmacokinetics of a drug, favorably adjusting the biodistribution in the body and releasing the active agent in a controlled and/or triggered manner. All these systems require the careful design of the polymeric carrier and there are number of requirements demanded of macromolecules before they can be considered for use in drug delivery. The obvious characteristics include good aqueous (plasma) solubility, biocompatibility and lack of immunogenicity. For most polymer therapeutics, multifunctionality is also essential to provide multivalent binding-release capacity for the attachment of medicinal payloads and/or targeting moieties.

Clearly, small-molecule drugs with a narrow therapeutic range (*i.e.*, a small difference between the minimum toxic dose and the minimum therapeutic dose) have the most to gain from conjugation to macromolecular carriers. Examples of such drugs, for which the side-effects are often dose-limiting, include anticancer drugs, antirheumatic and immunosuppressive agents [4]. Polymer therapeutics are, however, not limited to such substances and macromolecules have indeed been successfully used to aid the administration of a wide-range of substances, including a number of conjugates which have reached market or are currently in advanced clinical trials (summarized in [10]). The delivery of anti-cancer agents using polymeric carriers has been particularly intensely researched in recent years and significant gains have been made in terms of improving the often poor solubility, pharmacokinetics and severe side-effects of anti-cancer drugs [4,10]. With respect to cancer drug conjugates, the most important features include an enhanced aqueous solubility, reduced renal clearance and thus increased plasma half-life time and a passive tumor accumulation due to the enhanced permeability and retention (EPR) effect [11].

A high molecular weight is clearly a necessity when reduced renal clearance and increased plasma-circulation times are desired of the polymer-drug conjugate. However, the long-term use of non-degradable high-molecular weight polymers above the renal clearance limit (often reported to be around 30–50 kDa—the hydrodynamic radius of polymers with the same molecular weight but different chemical make-up and/or architecture can vary hugely and one should be cautious when comparing the molecular weights of different polymers) is of concern and can lead to deleterious accumulation [12,13]. This is especially so if, as for example with anti-cancer drug delivery, high amounts of polymer and repeated doses are required. There is, therefore, a pressing need to develop polymers with controlled biodegradability that can be repeatedly administered without prolonged administration in the body [3,12]. The vast majority of polymers investigated as carriers to-date are biopersistent polymers such as polyethyleneglycol (PEG) and poly(N-(2-hydroxypropyl) methacrylamide) (HPMA) which, despite showing excellent biocompatibility are inherently non-degradable and need to be used at molecular weights below the renal clearance limit. However, as explained earlier, a high molecular weight is often desirable for the enhancement of the pharmacokinetics. Furthermore, even polymers with molecular weights below the renal clearance limit run the risk of lysosomal accumulation leading to the induction of lysosomal storage disease [12]. It is therefore desirable, that polymers used for drug delivery applications not only degrade under physiological conditions, but degrade to small molecules rather than just to break up into oligomeric parts just below the renal clearance limit [13]. Although degradable units and/or linkages can be added to most synthetic polymers, synthetic polymeric carriers with the required structural control and high functionality to be used in polymer therapeutics are less common. The currently leading synthetic polymers under investigation for this purpose would appear to be polyglutamic acid and polyacetals [13] as well as dendritic polyesters [14].

Both biodistribution [15,16], as well as cell-uptake [17] are size dependent features and thus the ability to precisely control the size of the macromolecular carrier is essential. Studies have also shown that polymer architecture is of critical importance as it has a decisive influence on the biodistribution of the macromolecule [18,19]. It has, for example, been shown that an increased number of arms of the macromolecular carrier enhances blood circulation times [20], whereas rigid, elongated polymers show significantly enhanced tumor uptake compared to their less rigid counter-parts [21]. It becomes clear, that the precise control of polymer architecture is crucial for the success of macromolecular drug-delivery. Required are not only polymers with precisely controlled and tailored molecular weights, but also with a variety of controlled architectures.

The requirements of polymeric carriers for the delivery of macromolecular agents (e.g., proteins, DNA), in terms of control of molecular weight, solubility and biocompatibility, are similar to a large extent to those which govern the delivery of small-molecules via polymer conjugates, as detailed above. For example, polymers with controlled molecular weights and non-polydisperse structures are essential to enable control of structure–activity correlations between the carrier and the macromolecular therapeutic agent [22]. Furthermore, biodegradable polymers can enhance the safety of the carriers and multifunctionality can be used to incorporate targeting ligands, enhancing the specificity, as well as the incorporation of surface shielding side chains [22]. In gene therapy (the delivery of DNA into target cells, where it can act as a pro-drug for the synthesis of a therapeutic protein [23]), a number of additional factors must be taken into account. When delivering nucleic acids via cationic polymers, the polyplexes formed (with negatively charged DNA) must be shielded from possible biological interactions in the circulation system [22], and there a number of challenges faced in the internalization of the DNA in to cells (see [24] for more details). Although generally viral vectors are viewed as having better gene-transfer efficiency, serious immunogenicity issues have meant the search for nonviral vectors based on synthetic polymers has become ever more important [24].

In this review, whilst keeping the demands outlined above in mind, we describe how the inherent properties of polyphosphazenes, coupled with new advances in their chemistry are being used to develop polyphosphazene-based polymer therapeutics.

## 2. Poly[(organo)phosphazenes]

**Figure 1:**
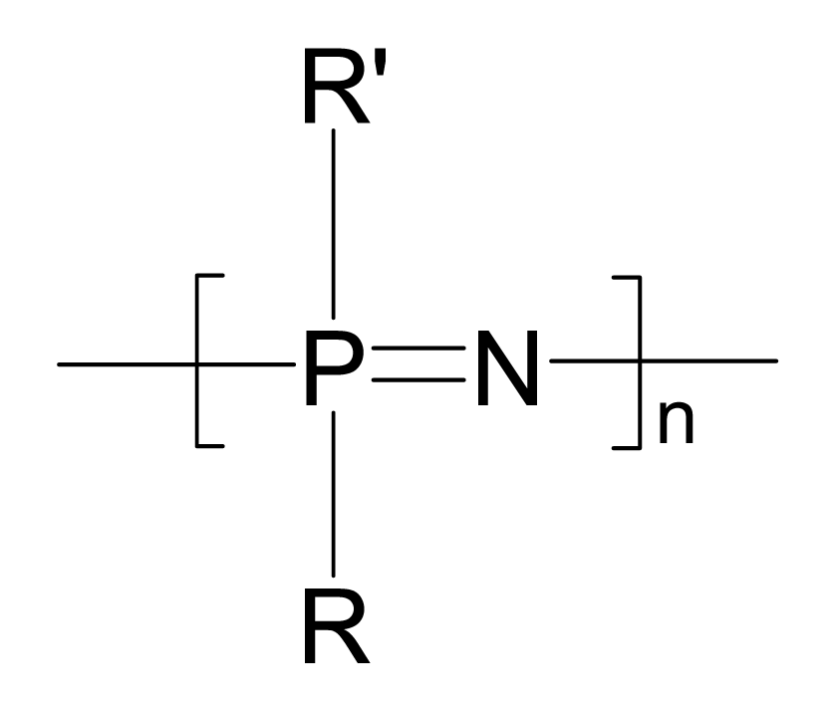
Generic structure of poly[(organo)phosphazenes].

**Figure 2:**
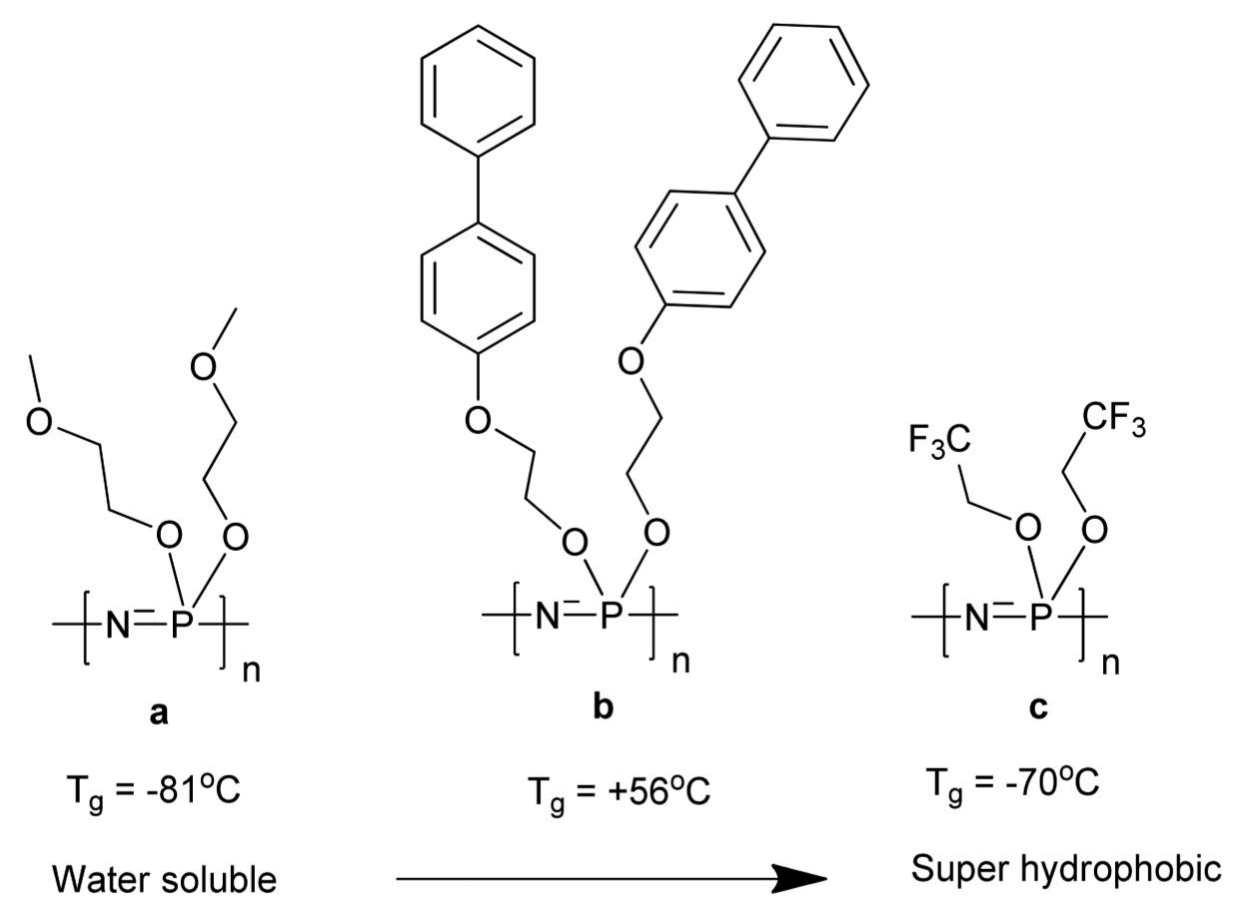
Some examples of poly[(organo)phosphazenes] with varied properties. (T_g_ values as reported in [25]).

Poly[(organo)phosphazenes] are inorganic/organic hybrid polymers. The polymer backbone consists of alternating phosphorus and nitrogen atoms and organic substituents are linked to the phosphorus atoms as side groups (Figure 1). The major precursor, polydichlorophosphazene, is extremely hydrolytically unstable but can be readily substituted with nucleophilic substituents to give a wide range of stable poly[(organo)phosphazenes] with an extremely wide range of properties [25]. The properties of the resulting material are highly dependent on the side-substituents and their ratios, for example, a difference in T_g_, of more than 130 °C is observed upon the addition of a biphenyl group to the ethylene oxide substituted polymer (Figure 2a,b).

This synthetic flexibility and versatile adaptability has resulted in a vast number of materials with a wide array of applications [26], ranging from water soluble polyphosphazenes [27] to superhydrophobic polymers [28] (Figure 2c). Furthermore, the R groups do not have to be identical; this offers the ability to add mixed substituents to control the functionality and the properties of the polymer. For example, a combination of recognition and release sites [29] or combining hydrophobic and hydrophilic properties to give amphiphilic polymers [30] or the substitution with catalytic units and solubilizing entities [31]. Depending on the side groups applied, polyphosphazenes can be bioerodible, which opens up routes for uses in tissue engineering and drug delivery [32-34]. Furthermore, the unique multiplicity of polydichlorophosphazene, with two functional groups per repeat unit, enables insertion of potentially high density of the required functional groups to the polymer backbone.

### 2.1. Synthetic Methods

**Figure 3:**
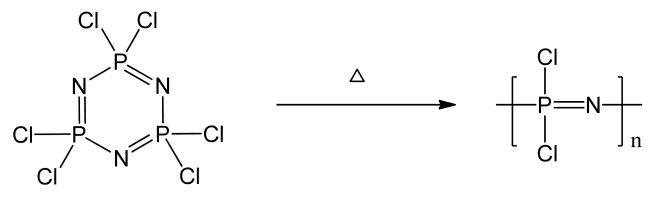
Polydichlorophosphazene by ring opening polymerization.

The most developed and widely-used method for the synthesis of polydichlorophosphazene is the thermal ring-opening polymerization of hexachlorophosphazene (Figure 3) (reviewed in detail in [25,35]). This can be carried out either in vacuum [36] or in high boiling solvents [37-39]. When the ring-opening reaction is applied, there is limited to no control over the molecular weight and thus the polymers generally have high molecular weights (M_w_ > 10^6^ daltons) and broad polydispersities [25]. Molecular weight control can be achieved by the use of catalysts such as OP(OPh)_3_/BCl_3_ or BCl_3_ [40], and also anhydrous aluminum chloride (2%–10%) [41]. This latter method is easy and convenient to perform and widely used but high temperatures are still required and polydispersity is broad (3.2–4.9), as is the case for the condensation polymerization of Cl_3_P=(O)Cl_2_ [42]. Importantly, a kilogram scale synthesis for manufacture of stabilized polydichlorophosphazene via ring-opening polymerization has been developed [43]. This process has been used for its reproducible manufacture for clinical studies.

**Figure 4:**
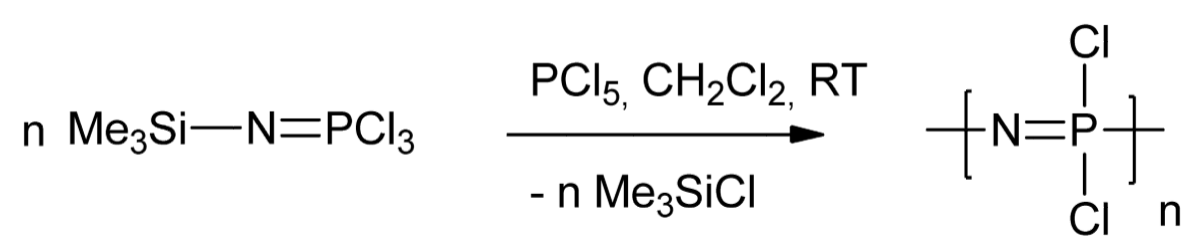
The living cationic polymerization route to polydichlorophosphazene.

A key development in the synthesis of polyphosphazenes was the room temperature, living cationic chain growth polymerization of chlorophosphoranimine (Figure 4), pioneered by Allcock and Manners [42,44,45]. This route enables the synthesis of polyphosphazenes with controlled molecular weights and narrow polydispersities (1.1–1.4). The development of a living polymerization route to polyphosphazenes was a key advancement allowing access not only to polymers with controlled molecular weights, but also to a variety of different molecular architectures. The same groups showed that they could utilize this route to give block copolymers [46,47], tri-armed star polymers [48] and also dendritic polymers, through attachment of living polyphosphazenes to a PAMAM core [49]. It has also been demonstrated that it was possible to prepare block copolymers using this method [47,50,51], some of which were shown to self-assemble into micelles [52].

Telechelic polyphosphazenes have also been synthesized by the Allcock group [53-55]. Through the capping of polyphosphazenes with norbornenyl functional groups they were successfully able to prepare a series of bottle brush polymers upon performing ring-opening methathesis polymerizations on the end-group functionalized polyphosphazenes. In separate work, Matyjaszewski *et al*. prepared AB block copolymers with alkyl phosphoranimine monomers [56,57], as well as some work in which phosphazenes are used as a core or backbone for star and graft organic polymers [58]. A combination of atom transfer radical polymerization (ATRP) and living cationic polymerization of polyphosphazenes was used to prepare densely grafted star- and comb-shaped molecular brushes [59,60]. The controlled polymerization also opens avenues to supramolecular structures [61,62]. Despite these successful initial studies, when compared to the plethora of structures available for other polymers there remains only this handful of reports in the literature in this area and the potential of this route it has not yet been fully exploited.

### 2.2. Biodegradation

**Figure 5:**
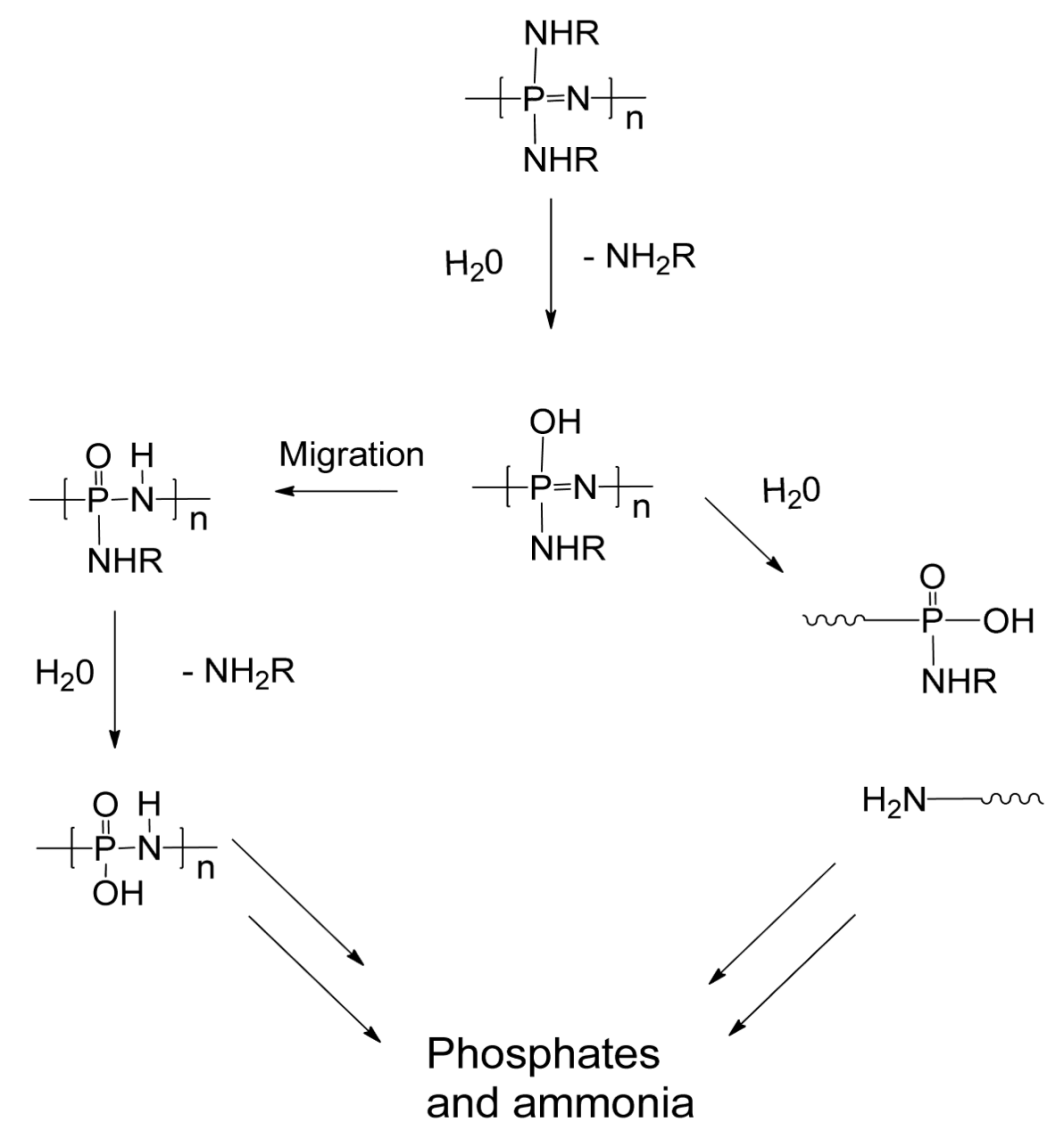
Non-catalyzed mechanism for the degradation of poly[(organo)phosphazenes].

A significant property of poly[(organo)phosphazenes], desirable for many biomedical applications, is the biodegradability of the polyphosphazene backbone. The main chain hydrolyses under physiological conditions to give, in addition to the corresponding side groups, low toxicity compounds including ammonia and phosphates (Figure 5) [25,63].

The hydrolytic stability can vary greatly, depending on the properties of the side-substituents and their hydrophilicity [64,65]. This property results in the ability to produce a wide spectrum of polymers with differing rates of degradation [66]. For example, hydrophilic amino substituted polyphosphazenes are known to be hydrolytically unstable, whereas substituting with hydrophobic, alkoxy side groups can produce extremely hydrolytically stable polymers. Furthermore, a variety of mixed substituents can be used, further allowing the fine tuning of the rate of hydrolysis. Such versatility is unrivalled in polymer chemistry.

**Figure 6:**
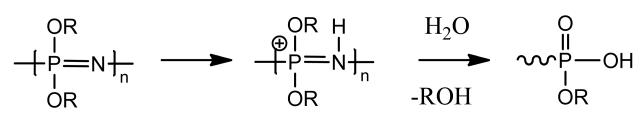
Acid catalyzed degradation mechanism.

Although substituents attached via an alkoxy group are generally expected to be less-susceptible to hydrolysis, as a result of the relative stability of the P-O-R bond, some very important bioerodible polyphosphazenes are also based on alkoxy linkages. These include, poly[di(carboxylatophenoxy)phosphazene] (PCPP) [67], as well as ethylpyrrolidone [68], lactate/glycolate [69], glyceryl [70] and glucosyl [71] derived poly[(organo)phosphazenes]. It is thought that proton transfer to the backbone nitrogen from the carboxylic acid groups of the polymer may be responsible for this [67]. Indeed it is commonly observed that degradation of polyphosphazenes is accelerated in acidic media (Figure 6) [66,72] and that the protonation of the phosphorus atom is responsible for this [73]. For some polymers, in particular polyphosphazenes substituted with amino acid esters or tertiary amino groups, an intramolecular catalysis mechanism has been proposed to explain the enhanced rate of degradation [74,75].

**Figure 7:**
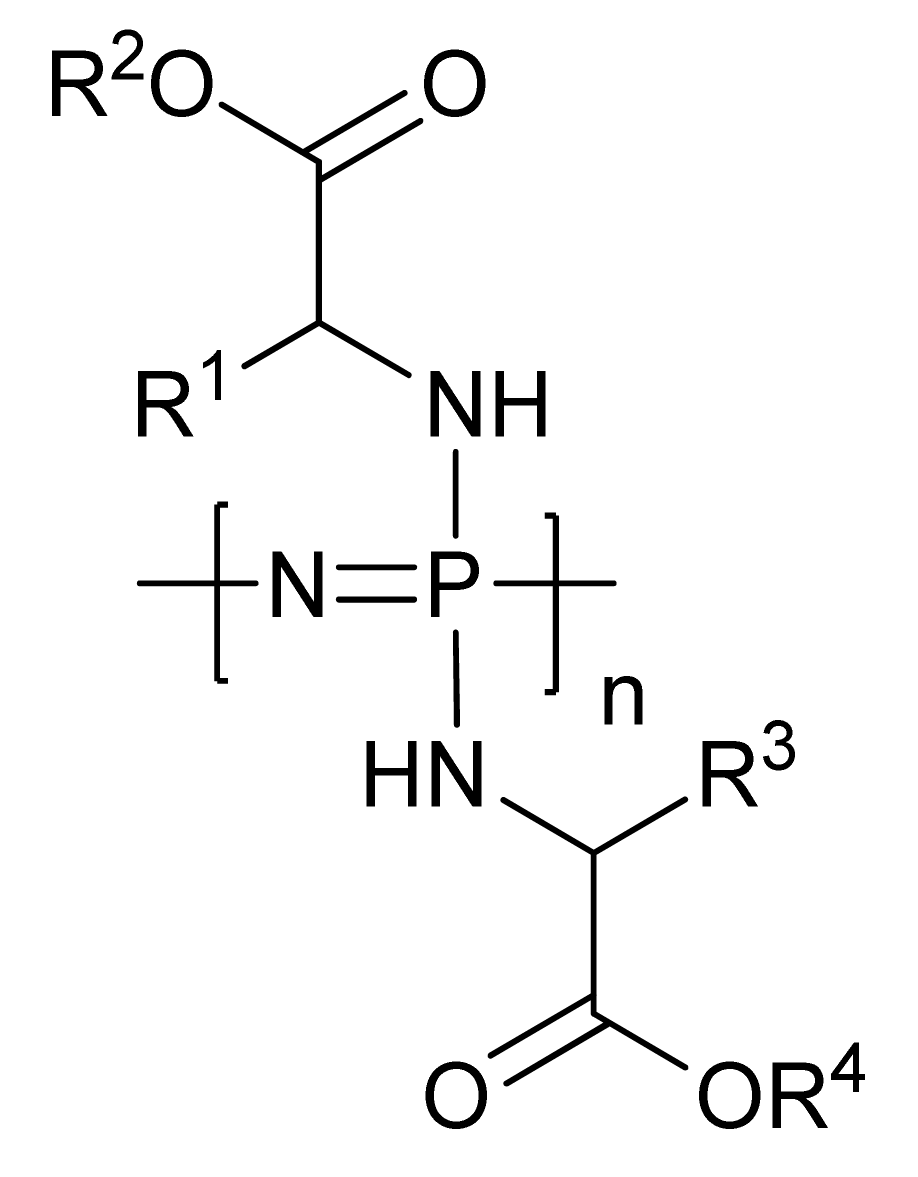
Generic structure for amino acid ester substituted poly[(organo)phosphazenes]. R_1_–R_4_ can be varied to give a range of poly[(organo)phosphazenes] with tailored degradation rates.

Poly[(organo)phosphazenes] based on amino acid esters (Figure 7) (also dipeptides and depsipeptides) have been particularly well investigated (see [66] and references therein). For amino acid ester derived polyphosphazenes, the nature of the α-position is observed to exert a significant influence on the rate of degradation of the polymer and hence variation of the attached amino acid can be used to tailor the degradation properties of the polymer [66]. Also, adding sterically large groups can shield the backbone from hydrolytic attack and thus delay hydrolysis. A commonly observed trend for the hydrolytic stability of the ethyl esters of the following amino acids is: glycine < alanine < valine < phenylalanine [64,75]. Amino acid esters are required, as opposed to free acids, due to the chain degradation and/or cross-linking that would be caused by the presence of carboxylic acid groups during substitution reactions. Subsequent deprotection to the free acid renders the polymers extremely hydrolytically instable [66]. The use of ethyl esters, as oppose to benzyl, butyl *etc.*, ensures the biocompatibility of the degradation products. At the same time, varying the bulkiness and hydrophobicity of the ester groups also alters the rate of hydrolysis, specifically; Me > Et > tBu > Bz [75]. The impact of such subtle changes in structure, coupled with wide availability of amino acid esters, makes such substituents a particularly simple route to access polymers with a wide range of degradation rates, ranging from years, in the case of phenylalanine ester, to days for glycine ester substituted polymer (for solid samples). Furthermore, through the use of mixed-substituents of different amino acid esters and/or hydrophobic co-substituents, it is possibly to further fine-tune the desired rate of degradation (e.g., in [76]). The hydrophilicity/ hydrophobicity of the side group substituents, and thus presumably access of H_2_O to the polyphosphazene backbone, also plays a significant role in the degradability of the polymers. This is expected to also play a role in the trend for the degradability of the amino acid ester substituents described earlier.

As mentioned earlier, hydrolytic breakdown is also known to be accelerated in acidic media [73,77,78]. This effect could be utilized to prepared controlled drug release systems. Interestingly, blends of poly[bis(ethyl glycinate)phosphazene] with polylactic-co-glycolic acid (PLAGA) were reported to have enhanced rates of degradation due to the action of PLAGA acidic degradation products on the polyphosphazenes backbone. The basic degradation products of the polyphosphazene are also reported to neutralize the highly acidic degradation products of the PLAGA component [79].

## 3. Applications in Drug Delivery

By far the most investigated application for bioerodible polyphosphazenes is their use a scaffolds for tissue engineering [76]. The tailorable mechanical and degradation properties can be used here to full effect to prepare mechanically stable materials with the required functionality and rate of degradation. This is, however, not the focus of this review and this is reviewed in detail elsewhere [80,81]. One of the key components of advanced materials for tissue engineering is the controlled release of growth factors and/or drugs from the matrix to support. For example, recently the controlled release of the antioxidant ferulic acid from polyphosphazenes based tissue scaffolds was described to protect cells from free radical damage [82]. It follows that a whole host of combinations can be imagined in which drug molecules can be either covalently attached to, or encapsulated into, bioerodible polyphosphazenes. Indeed it is over 30 year since Allcock showed how the slow hydrolysis of the polyphosphazene backbone could result in the controlled release of covalently bound anesthetics [83]. The following section attempts to highlight some of the key developments in the use of polyphosphazenes for drug delivery.

### 3.1. Matrix Encapsulation

Early investigations involved the release of drugs from matrices of amino acid ester and/or imidazole based poly[organo)phosphazenes] [63]. For example, variation of the ratios of the hydrolysis enhancing imidazole units in poly[(imidazoyl)(methylphenoxy)phosphazene] was used to deliver the model drug progesterone and also bovine serum albumin at a constant rate in a rat subcutaneous mode both *in vitro* and *in vivo* [84]. Similar materials were used to investigate the intra-articular administration of the anti-inflammatory drug colchicine [85]. Polyphosphazenes with imidazolyl (I-PPHOS) or ethyl glycinato (EG-PPHOS) side chain substituents were tested and colchicine release of 20% for I-PPHOS and 60% for EG-PPHOS was observed over the 21-day period.Similar results were obtained for preparation of naproxen sustained release systems [86]. *In vivo* studies detected after 28 days a 78% inhibition of arthritic oedema after subcutaneous administration of the matrices, whereas daily oral administration of naproxen resulted in only 29% inhibition. Upon optimization of the polymer composition, materials that maintained a sufficient level of naproxen in plasma for up to 400 h could be attained [87].

Protein release from degradable polyphosphazene matrices has also been investigated [88]. For example, insulin loaded degradable poly[(p-methylphenoxy)(ethylglycinato)phosphazene] matrices have also been reported [89]. As also reported for many polyphosphazenes, a pH dependent release of insulin was observed, with a lower pH resulting in an increased in rate of release. Interestingly, the loading of the protein had a decided effect on the rate of degradation, presumably due to the increased hydrophilicity of the matrix loaded with higher amounts of the protein. Insulin loaded microspheres have also been reported based on phenylalanine ethyl ester and imidazole substituents at a molar ratio of 80/20 [90]. The authors reported that a subcutaneous administration to diabetic mice prompted a decrease in glucose levels which was sustained for 1000 h and stimulated anti-insulin antibody production, which was observed to increase steadily over an 8 week period [90].

**Figure 8:**
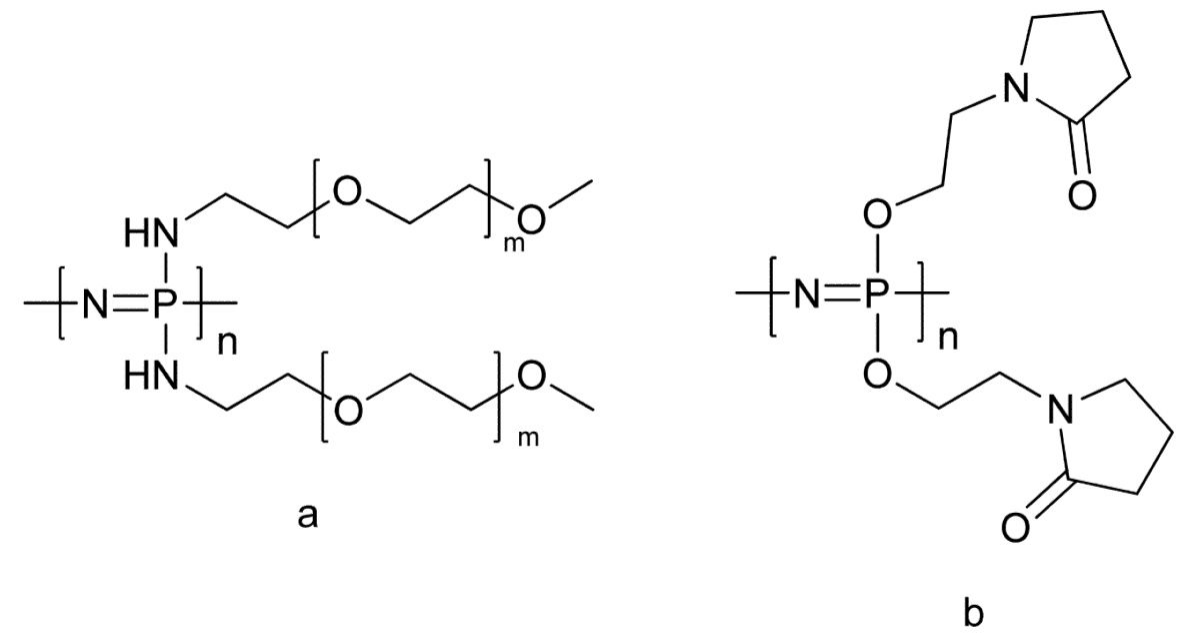
Polyphosphazenes substituted with (**a**) monomethoxy amino PEG and (**b**) N-ethylpyrrolidone side-substituents.

The large organic component of most poly[(organo)phosphazenes] means that the phosphorus-nitrogen backbone is shielded and thus polymers take on the chemical properties of the organic substituents. With this in mind, an important group of degradable polyphosphazenes for drug delivery may prove to be polyphosphazenes with N-ethylpyrrolidone groups attached (Figure 8b) [78]. Many commonly used solubilizers such as polyvinylpyrrolidone (PVP) and PEG are non-degradable and thus replacement with degradable analogues may offer a future alternative. In a similar vein, oligomeric PEG chains attached to the polyphosphazene backbone (Figure 8b) offer biodegradable versions of these commercially important polymers biomedicine (e.g., in [91]). In a similar fashion, glycolic and lactic acid derivatives have also been prepared, the phosphate-ammonia buffer produced by the degradation of the backbone can help mediate the acidity of the acids [79].

**Figure 9:**
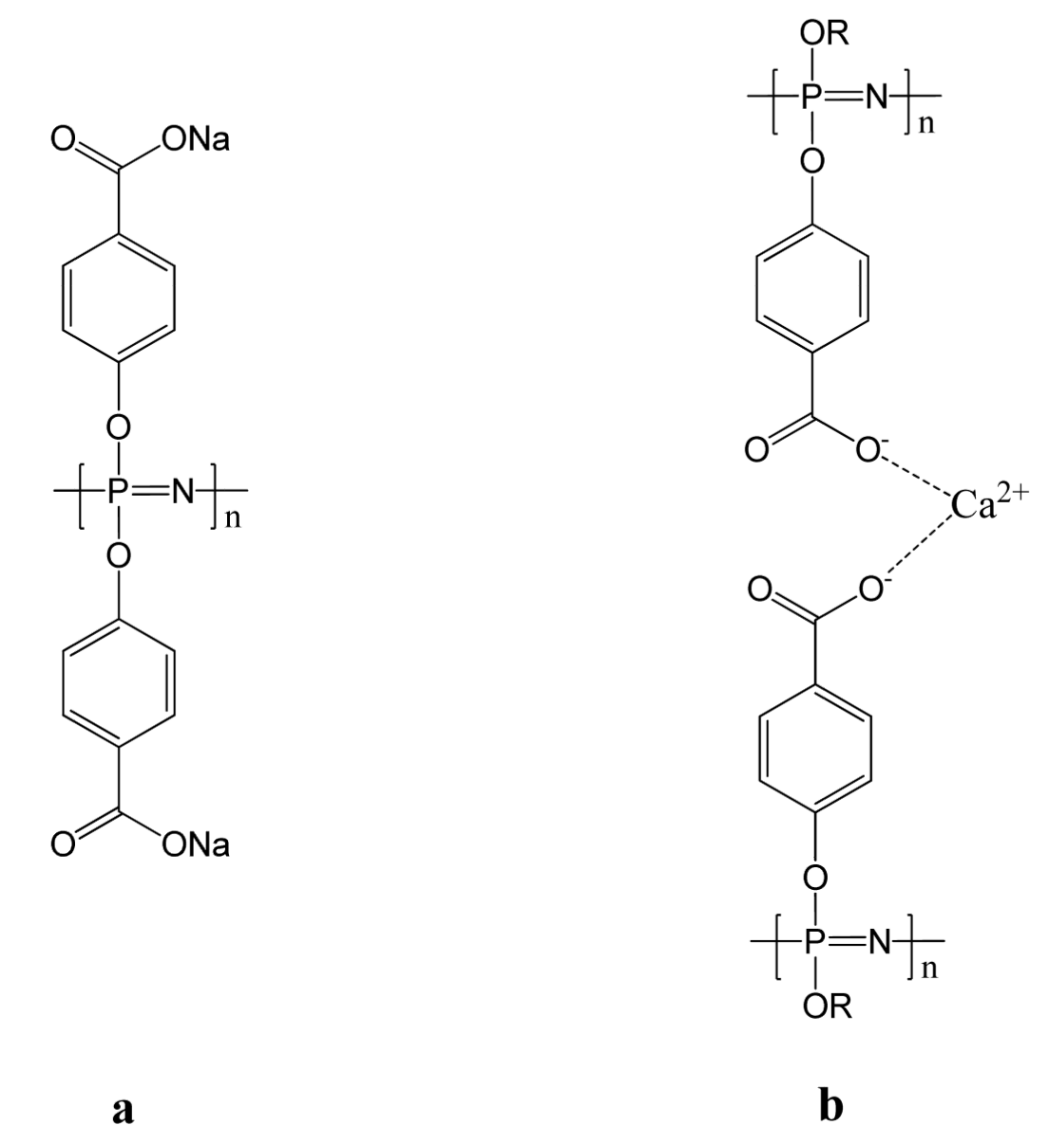
(**a**) Water soluble sodium salt of PCPP and (**b**) the calcium cross-linked calcium.

A further important group of polyphosphazenes are carboxylates and in particular the phenoxy carboxylate polymer poly[bis(carboxyphenoxy)phosphazene] (PCPP). Whereas the free acid is not water soluble, its sodium salt has excellent water solubility (Figure 9a). Furthermore, through adding the divalent salt calcium it can be cross-linked to give hydrogels (Figure 9b) [92]. Through the replacement of some of the sodium ions with calcium ions, cross-linked microspheres can also be produced. This system has been used for the encapsulation and controlled release of cells [93,94], proteins [95] and vaccines. The use as vaccine carriers is particularly promising and will be discussed in Section 3.6. PCPP degrades slowly in aqueous environments [67] and, as with all polyphosphazenes, this can be tailored through addition of degradation sensibilizing groups (see Section 2.2).

### 3.2. Anti-Cancer Drug-Conjugates

Simple, implantable matrix devices [96,97] and/or injectable microspheres [98] containing anti-cancer drugs have been also been developed for degradable polyphosphazenes. However, the unique multiplicity of polydichlorophosphazene enables the simple covalent bonding of drugs and/or functional groups for drug loading to the polymer backbone. This is particularly desirable as drug molecules covalently bound to their macromolecular carriers are known to be more effective transporters than systems in which the drug is supported via secondary interactions (e.g., micelle-based systems) whereby significant leakage is often observed [7,99]. Furthermore, the unique multiplicity of polydichlorophosphazene can be exploited to further add tumor-specific targeting ligands [5] to direct the drug to the site of action, as well as to add biocompatible, plasma-soluble groups (e.g., PEG) to enhance solubility and to produce a “stealth-effect”, effectively disguising the drug molecules and thus minimizing the immunogenicity [100].

**Figure 10:**
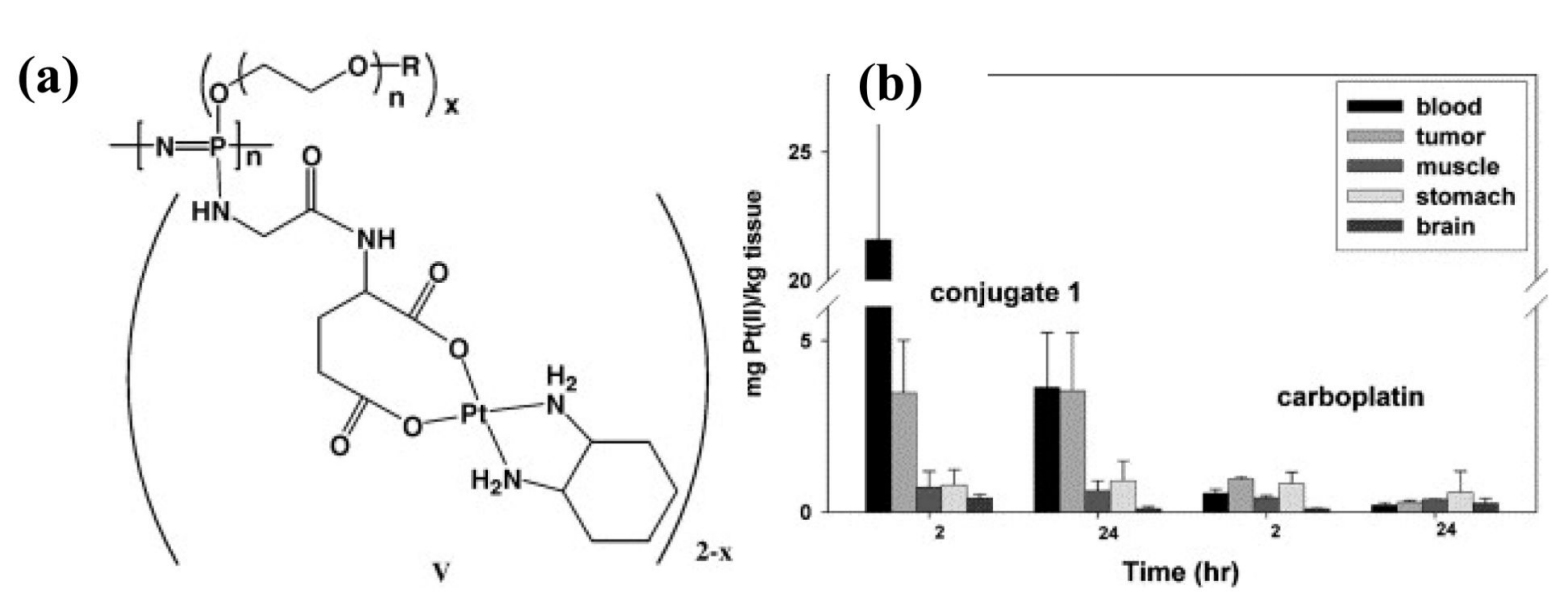
(**a**) An example of a polyphosphazene–platinum (II) conjugate. (**b**) A typical biodistribution profile of the polyphosphazene–platinum(II) conjugate showing enhanced retention of the polymer-conjugate in the blood and tumor compared to the analogous small drug carboplatin. Reprinted from [106], with permission from Elsevier.

The earliest anti-cancer drug conjugates in which the drug is covalently bound to the carrier were reported by Allcock and coworkers with polyphosphazenes in the 1970’s, long before the discovery of the EPR effect or controlled polymerization techniques [101,102]. The platinum complex K_2_PtCl_4_ was complexed to poly[bis(methylamino)phosphazene] to give a conjugate drug. No release of the platinum complex was observed and hence a low anti-tumor activity was observed for *in vivo* tests [101]. Some 20 years on, Sohn *et al*. were able to prepare water-soluble platinum II conjugated polyphosphazenes [103]. After some optimization of these polymer-conjugates [104] including replacement of the methylamine substituents with oligomeric polyethylene glycol groups, as well as optimization of the spacer groups [105], a polymer was selected for preclinical studies (Figure 10a). Excellent antitumor activity was also shown for these polymer-conjugates tested against human cancer cell lines [106] and furthermore they are reported to be biodegradable but no data is shown. The tumor uptake due to the EPR effect was observed to be around 10 times that for carboplatin (Figure 10b). Attempts to quantify the difference in EPR effect between the various conjugates showed only small differences, with a maximum tumor/tissue ratio for the polymer with an M_w_ = 62,800, R_h_ =11.4 nm [106]. However, polydispersities are not reported for the polymers used and since the ring-opening polymerization of hexachlorophosphazene is the reported synthetic method, it can be anticipated that the polydispersities are large which would have a significant effect on the biodistribution.

**Figure 11:**
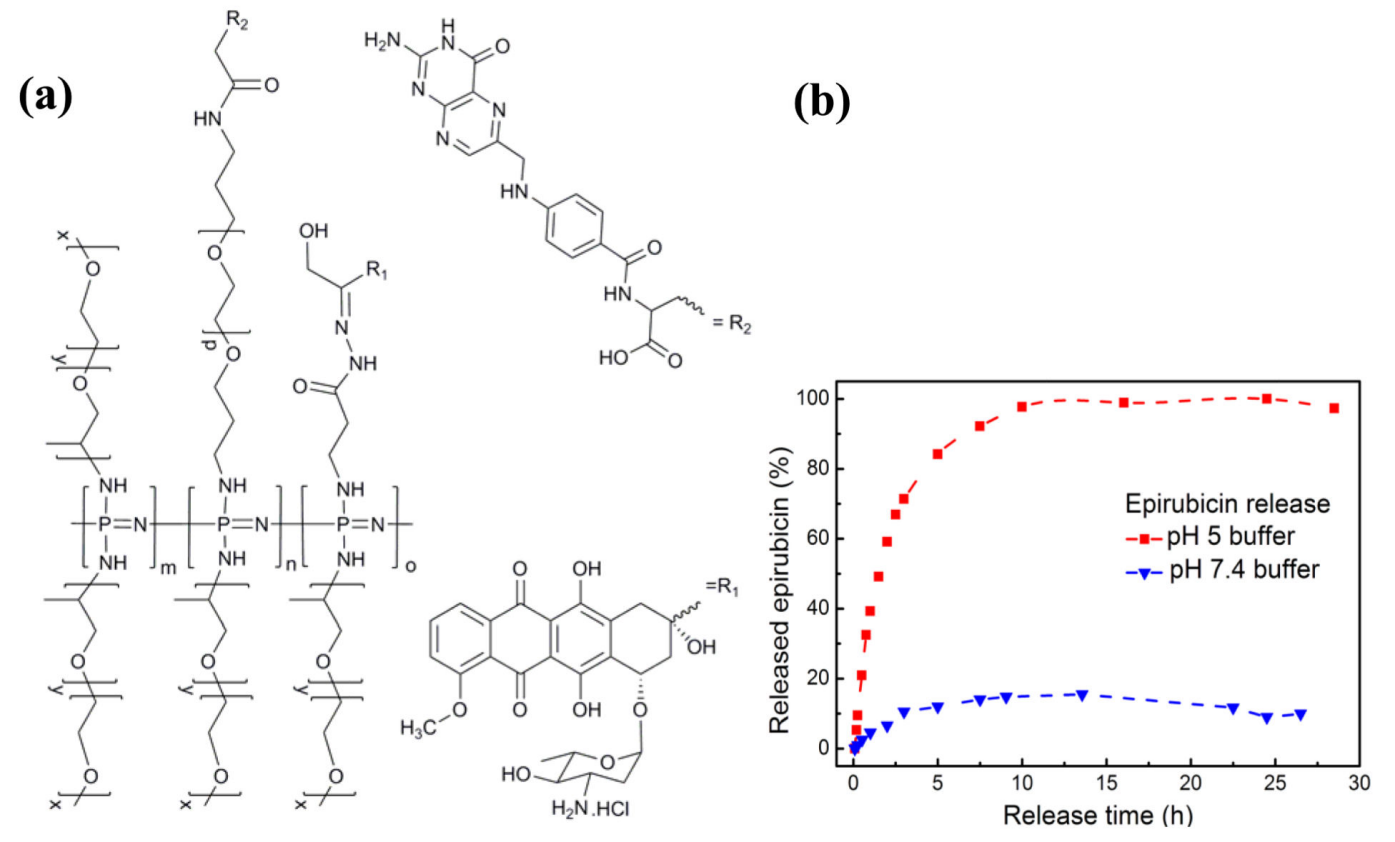
(**a**) Water soluble polyphosphazene loaded with a folic acid targeting ligands and epirubicin via a pH labile hydrazone bond. (**b**) Release of epirubicin from the hydrazone-linked polyphosphazene pH 5 (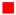) and pH 7.4(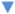). Originally published in [29] and reproduced by permission of The Royal Society of Chemistry.

Polymers for drug delivery with narrow polydispersities and controlled molecular weights have recently been prepared using the living cationic polymerization method [29]. The anticancer drugs epirubicin and doxorubicin were bound to the polyphosphazenes via a pH labile hydrazone linker and the remaining chlorine atoms replaced with polyalkylene oxide chains. The drug was discharged from the carrier in a pH controlled in a burst release and decoupled from biodegradation, *i.e.*, release was not dependent on erosion of the polymer backbone (Figure 11). Targeting ligands could also be added to the polymeric carrier and the rate of biodegradation could be controlled by the addition of amino acid ester cosubstituents. *In vitro* tests showed excellent cytotoxicity whilst no toxicity of the free polymer was observed [107]. Similar polymers with the photoactive drug hypericin covalently bound to the polyphosphazene backbone have also been prepared with narrow polydispersities via the living polymerization method [108]. The polymers were also shown to maintain the phototoxicity of the free drug whilst significantly enhancing its solubility and represent promising candidates for polymer assisted delivery for photodynamic therapy.

### 3.3. Self-Assembly

**Figure 12:**
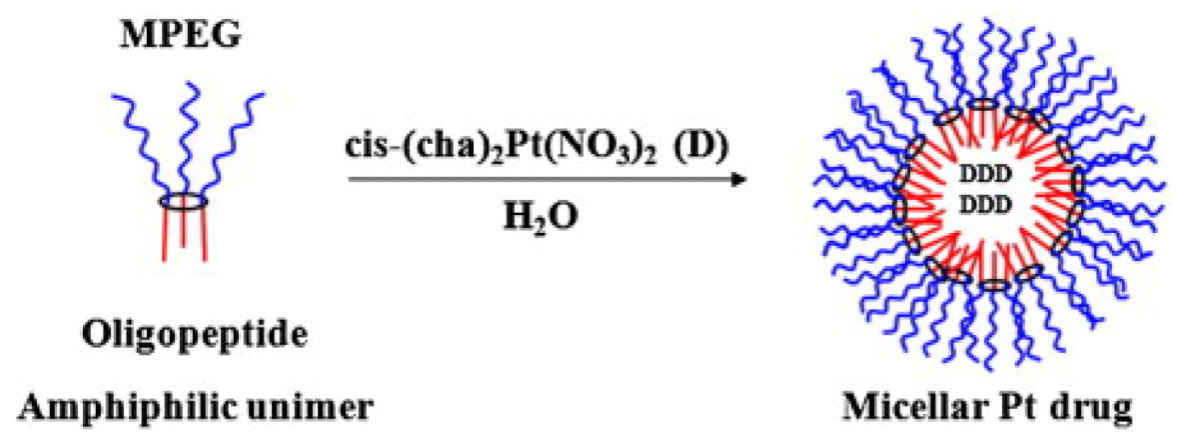
Conceptual diagram for the encapsulation of docetaxel in a cyclotriphosphazene amphiphilic micelle [NP(MPEG750)(GlyPheLeu)_2_Et]_3_. Reprinted from [115], with permission from Elsevier.

Clearly, any conjugation chemistry which can be applied to the phosphazene trimer hexachlorotriphosphazene can be applied to the linear polymer, and indeed *vice versa.* This has often been used to prepare model compounds for the chemistry on the high polymer [109]. Moreover, there are a number of examples of attempts to prepare pegylated conjugates with cyclotriphosphazene rings, in particular with platinum anti-cancer drugs [110-112]. Recently cyclotriphosphazenes have been reported in which the ring is substituted with hydrophobic oligopeptides and MPEG chains in various ratios. The amphiphilic oligomers were reported to self-assemble into stable micelles in aqueous solutions (Figure 12). Preclinical studies of the micelle-encapsulated, hydrophobic *cis*-(cha)_2_Pt(NO_3_)_2_ drug compound [113] showed extended blood circulation times in rats. Biodistribution studies showed tumor to tissue ratios of 4.03 2 h after injection and 4.67 24 h after injection and 6 times higher cellular uptake in tumor cells when compared to the free drug. It is also reported that micelle forming Pt anti-cancer drugs can also be covalently attached to the ring [114].

Micelles based on a similar chemistry of a PEG and oligopeptide substituted cyclotriphosphazene ring were also shown to self-assemble for the micelle-encapsulation of docetaxel [115]. Despite the excellent *in vivo* efficacy of docetaxel, encapsulation did not improve its plasma half-life and pharmacokinetic profile when compared to the free drug. Recently a similar cyclotriphosphazene derivative substituted by tryptophan ethyl ester groups, namely, hexa-[p-(carbonyl tryptophan ethyl ester) phenoxy)] cyclotriphosphazene (HEPCP), was reported to self-assemble into nanoparticles. The obtained nanoparticles were reported to be highly thermally stable and exhibit strong fluorescent emission [116].

Unlike the linear polyphosphazene chain, cyclic trimer itself is restricted in size and does not usually degrade readily, which could be a disadvantage when preparing biodegradable materials. Micellar structures, however, can also be prepared from linear amphiphilic diblock polyphosphazenes, prepared by the living cationic polymerization route, although initially prepared micelles required relatively high critical micellar concentrations [46,117]. Improved strategies include a “block-to” strategy, by which various organic blocks such as polypropylene glycol (PPG) [47] and PEG [51] can be linked to the living polyphosphazene chain. This involves coupling two individual polymers via reaction sites at the polymer ends, which although successful, has low coupling efficiency and difficult purification. A further improved strategy has recently been published using ATRP to grow hydrophilic blocks on a hydrophobic poly[(organo)phosphazene] to give a poly[bis(trifluoroethoxy)phosphazene]-co-poly[(dimethylamino)ethyl methacrylate] block copolymers. To the authors’ knowledge, drug delivery using any of these polymeric micelles has not yet been investigated.

### 3.4. Thermosensitive Polyphosphazenes

Poly(*N*-isopropyl acrylamide) (PNIPAm) oligomers have been grafted onto polyphosphazenes [118] with the hydrophobic glycine ethyl ester as cosubstituent in a random substitution pattern. These polymers show a temperature-triggered self-aggregation to micellar structures with a lower critical solution temperature (LCST) around 30 °C [30]. It has also been shown that, at lower temperatures the hydrophobic drug ibuprofen can be solubilized into polymeric aggregates (nanospheres) [119]. The authors report that drug release from such nanospheres must occur mainly through diffusion from the polymer matrix, as the polyphosphazenes do not degrade significantly during the time-frame of the release experiments. Similar polymers containing ethyl tryptophan as a cosubstituent were also tested. *In vitro* and *in vivo* drug studies showed a prolonged plasma lifetime and a slow, diffusion controlled release [120].

**Figure 13:**
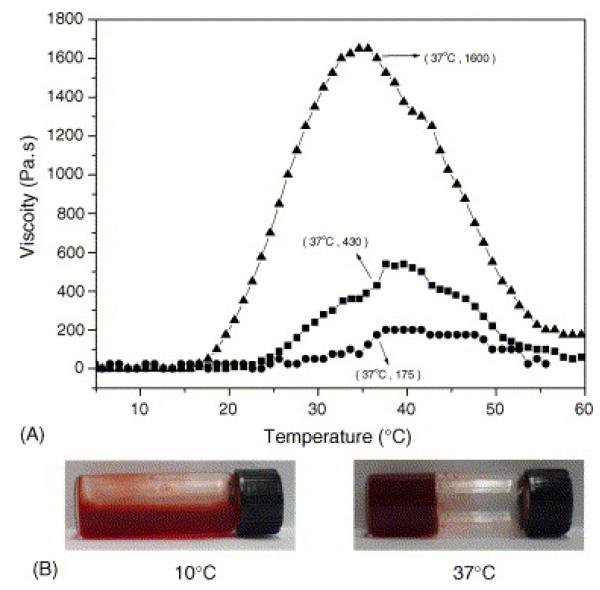
(**a**) Change of viscosity with temperature of polyphosphazenes cosubstituted with mPEG (M_n_ = 550) and isoleucine ethyl ester and glycyl lactate ethyl ester groups at polymer concentrations of 7 wt. % (●), 10 wt. % (■), and 15 wt. % (▼). (**b**) Reversible gelation behavior is observed at temperatures between 10 and 37 °C. Reprinted from [121]), with permission from Elsevier.

The ability to impart a thermosensitive response sol-gel transition as a function of temperature in polyphosphazenes also opens the opportunity to prepare injectable hydrogels (Figure 13). Thermosensitive poly[(organo)phosphazenes] bearing hydrophilic PEG chains (M_n_ 550) cosubstituted with hydrophobic isoleucine ethyl ester groups have been synthesized for the sustained delivery of the model drug 5-fluorouracil [122] and the anticancer drug doxorubicin [123]. Hydrolysis-sensitizing glycyl lactate ethyl esters were also added to enhance the rate of degradation of the polymer. The aqueous solution of poly[(organo)phosphazene] containing doxorubicin was transformed into a hydrogel at temperatures around that of the human body [121,124]. Similar polymers were used to enhance the bioavailability of the hydrophobic drug silibinin [125], which when intratumorally injected exhibited a good antitumor effect due to the sustained release of the drug. The ability to add multiple mixed substituents to polyphosphazenes was utilized to insert multiple thiol groups to the polymer, which can further improve the gel strength by chemical cross-linking of thiol groups with divinyl crosslinkers under physiological conditions [126].

The uniquely high functionality of polyphosphazenes has also been exploited to insert further cationic groups onto such thermosensitive hydrogels [127] for the sustained delivery of the negatively charged human growth hormone (hGH). This could be used to tailor the sustained delivery of hGH in both *in vitro* and *in vivo* investigations.

### 3.5. Polyplexes for Gene Delivery

**Figure 14:**
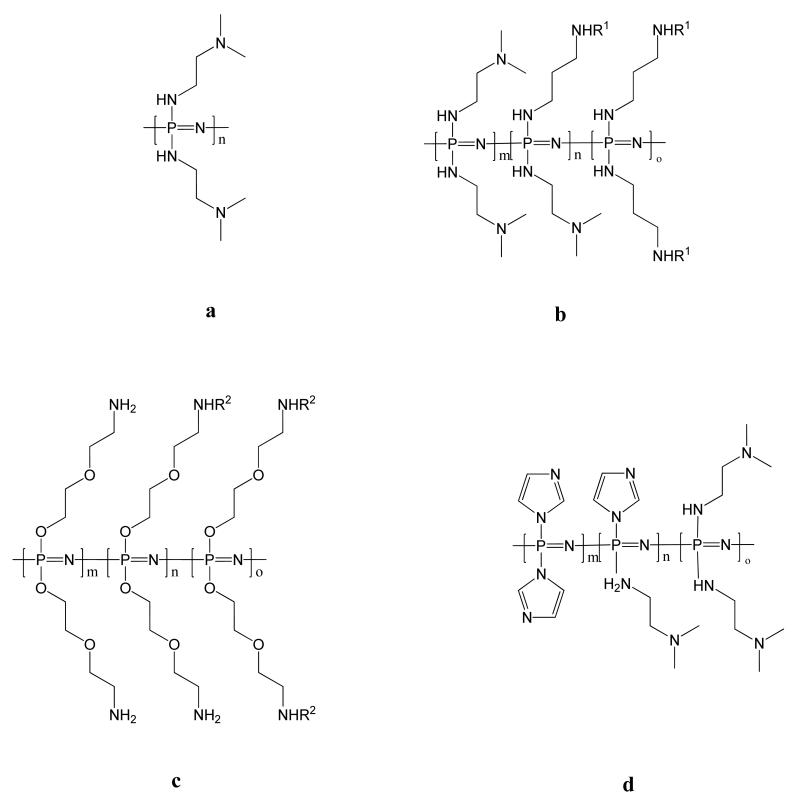
Structures of cationic polyphosphazenes tested for gene delivery. R^1^ and R^2^ can be varied for the attachment of, for example, tumor targeting ligands or pegylating units.

Gene therapy, the use of therapeutic nucleic acids, represents a highly promising route in the fight against cancer. Synthetic polymers are being used to attempt to address the difficulties in providing a specific and efficient intravenous delivery of genetic material to metastatic tumors [128]. The biodegradability and multifunctionality of polyphosphazenes can be utilized to prepare potentially safer alternatives to commonly used biopersistent cationic polymers such as polyethyleneimine (PEI) and polyamidoamine PAMAM. One of the first polyphosphazenes to be investigated for this purpose is poly[(2-dimethylamino ethylamino)phosphazene] (Figure 14a). This polymer is reported to degrade in physiological conditions with a half-life of around 24 days [74]. The polyphosphazene based polyplex bound plasmid DNA yielding anionic polyplexes that transfected COS-7 cells. The efficiency was reported to be comparable the non-biodegradable polymeric transfectant [poly(2-dimethylaminoethyl methacrylate), pDMAEMA] and the toxicity significantly lower. *In vitro* studies showed a much lower transfection efficiency compared to the well-known non-biodegradable linear polyethylenimine (PEI) [129]. However, the degree of tumor gene expression was shown to be significant enough for *in vivo* studies to be conducted. Remarkably, *in vivo* studies showed in contrast to PEI carriers, that gene expression was mainly limited to tumor tissue and as such polyphosphazene based polyplexes offer significant promise as gene transfection agents. Cosubstitution of the similar polyphosphazenes allows the potential tailoring of the polymeric carrier. For example, Yang *et al*. [130] prepared poly[(imidazole)(DMAEA)phosphazene] (Figure 14d), which they termed PDIP, and was reported to have enhanced gene transfer activity but lower cytotoxicity than DMAEA substituted polyphosphazenes and PEI polyplexes.

The multifunctional nature of polyphosphazenes has also been exploited to post-pegylate the polyplexes and also to incorporate tumor targeting folate ligands to the polyphosphazenes [27]. Folate ligands were also shown to enhance the cellular uptake of polyphosphazenes with primary amine substituents (Figure 14b,c). In a similar fashion polyphosphazenes with galactose moieties have also been prepared [131]. The authors reported selective gene expression for galactose decorated polyphosphazenes in the tumor and liver with comparatively little gene expression in other organs [131].

As explained earlier in this review, polymer molecular weight, and hence polydispersity, have a significant impact on the biodistribution of polymer carriers. For this reason, de Wolf *et al*. [132] used preparative size exclusion chromatography to fractionate polyphosphazenes, previously prepared by the ring-opening polymerization method (see Section 2.1). The authors were hereby able to reduce the polydispersities to 1.1–1.3. The living polymerization method would appear to be a more suitable method, if high molecular weight, cationic polyphosphazenes could be prepared. Recently cationic polyphosphazenes have been prepared via the living cationic polymerization, although the application was not aimed at gene delivery but their use as anti-bacterial agents [60].

Similar polyplex structures, with diisopropylamino (DPA) and PEG side groups cosubstituted in various amounts have also been investigated for their anti-tumor activity [133]. The ratios of DPA to PEG groups could be tailored so that in the endosomal pH range, the polymers showed enhanced membrane disruptive activity. Moreover, DPA-grafted polyphosphazenes were shown to significantly inhibit the drug efflux activity of P-glycoprotein (P-gp) on the plasma membrane of drug-resistant tumor cells and thus have potential for intracellular drug delivery applications, especially for the treatment of P-gp overexpressing, drug-resistant tumors.

### 3.6. Vaccine Delivery and Immunomodulation

**Figure 15:**
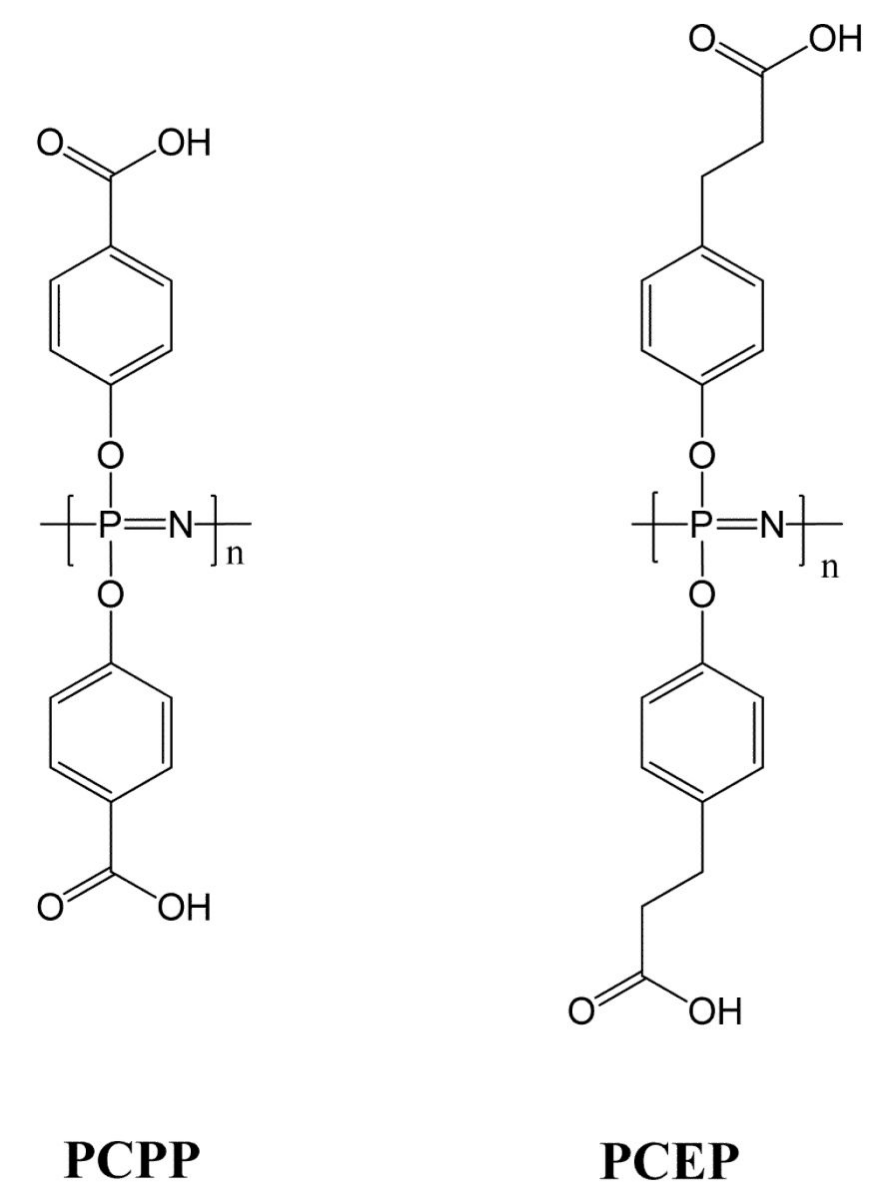
The most important polyphosphazenes most used as a basis for vaccine delivery vehicles are the sodium salts of poly[bis(carboxyphenoxy)phosphazene] PCPP and poly[bis(carboxyethylphenoxy)phosphazene] PCEP.

Due to the unique high density of functional groups and the flexibility of the backbone, charged poly[(organo)phosphazenes] are able to form water soluble polyplexes with antigens [88]. Furthermore, a number of studies have shown that polycarboxylate polyphosphazenes also act as potent immunological adjuvants. Vaccine adjuvants are substances that enhance adaptive immune responses to an antigen adjuvant [134]. Investigations have focused on the two high-molecular-weight, degradable polyphosphazenes poly[bis(carboxyphenoxy)phosphazene] (PCPP) and poly[bis(carboxyethylphenoxy)phosphazene] (PCEP) (Figure 15), both prepared by the ring-opening polymerization route. They have been shown to bind to a wide array of antigens. The application of these two polyphosphazenes in the delivery of vaccine antigens and immunotherapeutic agents have recently been reviewed in detail [135,136] and thus will only be summarized briefly here.

For PCPP, a wide range of *in vivo* tests have been carried out over the last 20 years on bacterial and viral antigens in a variety of animal models. Although the mechanism for the adjuvant activity is still not well understood [137], it is observed to significantly enhance immune responses when co-administered with a diverse array of bacterial and viral antigens. Limited but nevertheless successful clinical trials in humans have shown that a PCPP-vaccine formulation was indeed safe and immunogenic ([138], p. 53).

In recent years, PCEP has been the focus of intense investigations as it has been reported to possess adjuvant activity above and beyond that of PCPP (see [135] and references therein). Furthermore, in studies in mice, it has been shown to have significant advantages over the widely used adjuvant aluminium hydroxide (Alum) [135]. Potent adjuvant activity in large animals is also reported, with tests in sheep ([138], pp. 77–83) and more recently pigs [139] showing the effectiveness and safety of the use of PCEP. As well as molecular adjuvants, supramolecular and microsphere formulations can also be prepared in which the encapsulation of the antigen is followed by a controlled/sustained release [140]. These are thought to be particularly promising for the mucosal delivery of antigens. Such slow-release encapsulation systems use calcium-cross-linked PCPP microspheres in an analogous approach to that developed for the controlled release of encapsulated proteins such as albumin (see Section 3.1). Furthermore, it can be expected that polymer molecules released during degradation of the microsphere would also have an adjuvant effect of the individual polymers [88].

## 4. Conclusions

In terms of on-the-market applications, and indeed clinical studies, poly[(organo)phosphazenes] lag far behind many more established polymers for drug delivery applications. However, the number of promising *in vitro* and *in vivo* studies in a wide-range of therapies, detailed in this review, show the potential of poly[(organo)phosphazene] in drug and gene delivery applications. Recent advances in the chemical synthesis of the major precursor polydichlorophosphazene, not least the development of a robust living polymerization route, alongside the inherent high functionality of the phosphorus-nitrogen backbone, makes these extremely promising materials for this field. Moreover, the inherent biodegradability, which many examples have shown can be readily tuned, make them an extremely promising group of materials for drug delivery with a unique combination of properties, which without doubt warrant future investigation.
